# Test concordance and diagnostic accuracy of three serological assays for detection of anti-SARS-CoV-2 antibody: result from a population-based sero-epidemiological study in Delhi

**DOI:** 10.1186/s12879-022-07805-5

**Published:** 2022-12-07

**Authors:** Puneet Misra, Shashi Kant, Randeep Guleria, Mohammad Ahmad, Suprakash Mandal, P. K. Chaturvedi, Guruprasad R. Medigeshi, Suneeta Meena, Sanjay Kumar Rai, Anisur Rahman, Meenu Sangral, Kapil Yadav, Mohan Bairwa, Partha Haldar

**Affiliations:** 1 Centre for Community Medicine, All India Institute of Medical Sciences, New Delhi, 110029 India; 2 All India Institute of Medical Sciences, New Delhi, 110029 India; 3 WHO Country Office, World Health Organization, New Delhi, India; 4 Department of Reproductive Biology, All India Institute of Medical Sciences, New Delhi, 110029 India; 5 Translational Health Science and Technology Institute, Faridabad, 121001 India; 6 Department of Laboratory Medicine, All India Institute of Medical Sciences, New Delhi, 110029 India; 7 WHO, New Delhi, India

**Keywords:** Concordance, Serology, Serum, Antibody, SARS-CoV-2

## Abstract

**Background:**

Several methodological tests are available to detect SARS-CoV-2 antibody. Tests are mostly used in the aid of diagnosis or for serological assessment. No tests are fully confirmatory and have variable level of diagnostic ability. We aimed at assessing agreement with three serological tests: quantitative anti receptor binding domain ELISA (Q-RBD), qualitative ELISA (WANTAI SARS-CoV-2 Ab) and qualitative chemiluminescence assay (CLIA).

**Methods:**

This study was a part of a large population based sero-epidemiological cohort study. Participants aged 1 year or older were included from 25 randomly selected clusters each in Delhi urban (urban resettlement colony of South Delhi district) and Delhi rural (villages in Faridabad district, Haryana). Three type of tests were applied to all the baseline blood samples. Result of the three tests were evaluated by estimating the total agreement and kappa value.

**Results:**

Total 3491 blood samples collected from March to September, 2021, out of which 1700 (48.7%) from urban and 1791 (51.3%) from rural. Overall 44.1% of participants were male. The proportion of sero-positivity were 78.1%, 75.2% and 31.8% by Wantai, QRBD and CLIA tests respectively. The total agreement between Wantai and QRBD was 94.5%, 53.1% between Wantai and CLIA, and 56.8% between QRBD and CLIA. The kappa value between these three tests were 0.84 (95% CI 0.80–0.87), 0.22 (95% CI 0.19–0.24) and 0.26 (95% CI 0.23–0.28).

**Conclusions:**

There was strong concordance between Wantai and QRBD test. Agreement between CLIA with other two tests was low. Wantai and QRBD tests measuring the antibody to same S protein can be used with high agreement based on the relevant scenario.

## Background

Approach to test anti-SARS-CoV-2 antibody level is a valuable aspect of this COVID-19 pandemic response. This is helpful to understand the extent of past infection as well as level of immune response at individual level and for serological surveillance in a population [1, 2]. This can also be used as an aid of diagnosis for recent past infection and among the suspected COVID-19 patient having post-COVID illness [2, 3]. The antibody test should have high level of accuracy to capture wide group of population especially the asymptomatic group [4]. There are several type of serological tests that detect IgG and or IgM using different methodology e.g. enzyme linked immunosorbent assay (ELISA), chemiluminescence assay (CLIA), lateral flow immunoassay [5]. The target protein and the type of antibody captured by the test kit influences the proportion of positive samples in a group of population. Therefore these assays have variable level of sensitivity, specificity and concordance [6, 7]. Due to these variable accuracy, the interpretation of the serological tests result of different tests are challenging in the context of epidemiological as well as clinical scenario which necessitates requirement of further study [8]. The present sero-prevalence study had used quantitative anti receptor binding domain ELISA (Q-RBD), qualitative ELISA (WANTAI SARS-CoV-2 Ab) [9] and qualitative chemiluminescence assay (CLIA) [10]. We aimed to assess the test concordance of these test kits for the SARS-CoV-2 specific antibody detection.

## Methods

### Design and participants

This study was part of a multi-centric population-based, age- stratified prospective cohort study. The study population was from selected clusters of rural and urban area. Any participants with age more than equal to 1 year were included considering the practical difficulty of obtaining consent, blood sample among the infant age group. The participants not willing to give blood sample were excluded. In each area, all family members of ≥ 1 year age from the consecutive families were included from 25 randomly selected clusters assuming four participants per family. In rural area, individual village was considered as cluster whereas municipality ward was taken as cluster in urban area. We are reporting the baseline data only.

The data collection period was from March to September, 2021. Informed written consent was obtained from all the participants and the samples were collected maintaining all recommended precautions. The study was approved by the Institutional ethics committee of AIIMS, New Delhi.

### Procedure and setting

Participants of both urban and rural area were included. The urban area of Delhi state was a resettlement colony in South Delhi district. The area was mainly populated migratory people and of lower economic strata having a population of 36,000 as of 2021. The rural participants were from the rural area of Faridabad district of Haryana state which comes under Delhi National Capital Region (NCR). The area was the field practice area of the investigating institution. It had total 28 villages with a population of 101,000 in 2021. Out of the 28 villages, 25 villages were selected by probability to proportion to size sampling method. The data collection team consists of a research officer, nursing staff, lab technician and a field attendant. They were directed to start recruiting the participants from a randomly selected lane of a site preferably from the centre of the cluster. All the participants of aged more than equal to 1 year were approached from the consecutive houses. The rule of left were adopted to move at the end of the lane. After taking due consent and or assent, 5 ml of venous blood sample was collected in clot activator vials (yellow top with gel separator) from each participant. The participants were interviewed by trained staff to collect information related to basic socio-demographic factor, vaccination details etc. The serum was separated within 2 h of collection. The serum samples were transported to laboratory on the same day maintaining the cold chain temperature of 2–8 degree Celsius. In the laboratory, the sera were stored at -80 degree temperature until analysed.

### Outcome variables

Antibody level to SARS-CoV-2 virus in human serum was assessed quantitatively and qualitatively based on the type of test kit.

### Laboratory tests

Each of the serum sample was analysed by three different serological tests within seven days of separation of serum. The first test was a qualitative assessment of SARS-CoV-2 antibody by ELISA method using Wantai SARS-CoV-2 Ab ELISA kit. The second assay was quantitative estimation of the SARS-CoV-2 antibody by in house developed ELISA kit. The third test was qualitative assay by chemiluminescence method using Abbott laboratories (Table 1).

**Table 1:** Specification details of the three serological tests used to detect Anti-SARS-CoV-2 antibodies

Test specifications	WANTAI SARS-CoV-2 Ab ELISA^¥^	Quantitative anti-RBD ELISA (QRBD)^€^	Chemiluminescence assay (CLIA)
Manufacturer	Beijing Wantai Biological Pharmacy Enterprise Co., Ltd, China	In-house kit developed by Translational Health Science And Technology Institute (THSTI), India	Abbott Laboratories Diagnostics, USA
Principle	ELISA	ELISA	Chemi-luminiscence Assay
Type	Qualitative	Quantitative	Qualitative
Target protein	Receptor binding domain of spike protein	Receptor binding domain of spike protein	Nucleocapsid protein of SARS-CoV-2
Antibody captured	Total antibody (IgM + IgG + IgA)	IgG	IgG
Test sensitivity	94.5%	100%	Unknown^@^
Test specificity	100%	100%	Unknown^@^
Reference Value	≥ 0.19 OD = Positive < 0.19 OD = Negative	≤ 7.99 BAU^#^/ml = Negative > 7.99—12.0 BAU^#^/ml = Equivocal ≥ 12.0 BAU^#^/ml = Positive	< 1.4 S/C^$^ = Negative ≥ 1.4 S/C^$^ = Positive

^¥^WANTAI Severe Acute Respiratory Syndrome Corona Virus-2 antibody Enzyme-Linked Immunosorbent Assay

^€^Quantitative anti-receptor binding domain Enzyme-Linked Immunosorbent Assay

OD: optical density

^#^BAU = ELISA Unit

^$^S/C = Ratio of the mean chemiluminescent signal of sample and calibrator

^@^As mentioned in the kit information brochure

### Methodology for WANTAI SARS‐CoV‐2 Antibody ELISA

This was done by sandwich ELISA kit using a polystyrene microwell in two steps. In the first step, the serum of the participants were added into which the antibody got adhered to the wells. The free antibody was then washed away. The second step was to add recombinant SARS-CoV-2 antigen which was conjugated with Horseradish Peroxidase (HRP-Conjugate) was added. The antigen got bound to the antibody which was captured in the first step in the well forming a “sandwich” immune complex. This reaction was reflected by the yellow colouration once a colourless chromogen solution came in contact with the immune complex. Those sample having the absorbance to Cut-off ratio of ≥ 1.0 was accepted as positive.

### Methodology for QRBD

This was also done in a two-step approach. A 96 well polystyrene plate was coated with recombinant spike protein. The anti-RBD antibody in the participant’s serum reacted with the coated RBD antigen while extra unbound antibody was washed away. HRP-conjugated anti-IgG containing solution was added which bound to the human IgG bound to the RBD protein in the micro well. The reaction was captured by the blue to yellow colouration after adding Tetramethylbenzidine (TMB) and sulphuric acid (H_2_SO_4_). The colour intensity was measured by the absorbance of the solution by 450 nm of wave. The intensity of the colour was directly proportional to the amount of anti-RBD antibodies present in the serum.

### Methodology for CLIA

This was also a two-step immunoassay with chemiluminescent micro particle technology principle. Micro particle coated with SARS-CoV-2 antigen were combined with assay diluent followed by incubation. The antibodies present in the participant’s serum binds with the antigen coated micro particle. Anti-human IgG labelled with acridinium conjugate was added followed by pre-trigger and trigger solution. The test reaction was measured by system optics and expressed as relative light unit (RLU). The level of RLU was directly proportional to the amount of IgG. It was then compared to the calibrator RLU to determine the presence and absence of IgG antibodies against SARS-CoV-2.

### Data analysis

The data were extracted in Microsoft excel and analysed in STATA V12 statistical software. The qualitative test results of antibody were expressed as proportion whereas the quantitative level of antibody were expressed in mean value with 95% confidence interval. The test accuracy measure was estimated by sensitivity and specificity considering each test as the gold standard. Concordance were expressed by total agreement calculated between QRBD & CLIA, WANTAI & QRBD and WANTAI & CLIA and by Cohen’s kappa value for the agreement beyond chance.

## Results

The data collection period was from March to September, 2021 in Delhi urban and in rural area. The total collected sample was 3491 where 1700 (48.7%) was from urban area and 1791 (51.3%) from rural area. In the urban area 9.3% participants were under 18 years of age whereas in rural area it was 18.5%. Overall 44.1% of the participants were male with a slight higher proportion in rural area (46.8%). In urban area 94.0% participants were un-vaccinated whereas in rural it was 84.7% (Table 2). A total of 78.1% samples were sero-positive by Wantai, 75.2% by QRBD and 31.8% by CLIA tests respectively (Table 3). As per the QRBD test, the mean antibody level among positive group was 270.6 BAU/ml (SD = 424.3, 95% CI 253.5–287.8) and among negative group it was 2.4 BAU/ml (SD = 2.1, 95% CI 2.3–2.6). The sensitivity and specificity of QRBD in respect to WANTAI was 94.6% (95% CI 93.6–95.4%) and 94.3% (95% CI 92.3–95.8%). The sensitivity and specificity of CLIA in respect to WANTAI was 40.3% (95% CI 38.4–42.2%) and 98.4% (95% CI 97.3–99.2%) respectively (Table 4). The sensitivity and specificity of CLIA in respect to QRBD was 42.9% (95% CI 41.0–44.9%) and 99.0% (95% CI 98.1–99.5%) (Table 5). The total agreement between Wantai and QRBD was 94.5%, 53.1% between Wantai and CLIA, and 56.8% between QRBD and CLIA. The kappa value between these three tests were 0.84 (95% CI 0.80–0.87), 0.22 (95% CI 0.19–0.24) and 0.26 (95% CI 0.23–0.28) (Tables 4 and 5). The total proportion of positive test result was found almost similar among the population both in vaccinated and unvaccinated. This finding was consistent across all the three tests. When compared the seropositivity among the recipients of single or two doses of vaccine, it was seen that the proportion of positive tests were higher among those getting two doses of vaccine (Table 6).

**Table 2:** Distribution of the participants according to age, sex and vaccination status in both rural and urban area

Variables	Category	Urban (n = 1700) n (%)	Rural (n = 1791) n (%)
Age	< 18 years	157 (9.3)	331 (18.5)
	≥ 18 years	1543 (90.7)	1460 (81.5)
Sex	Male	699 (41.1)	839 (46.8)
	Female	1001 (58.9)	952 (53.2)
Vaccination	Vaccinated	98 (5.7)	273 (15.2)
	Un-vaccinated	1599 (94.0)	1518 (84.7)
	Unknown	3 (0.2)	0

**Table 3:** Proportion of qualitative result of the serum antibody by WANTAI, QRBD and CLIA tests

Test	Positive n (%)	Negative n (%)
WANTAI (n = 3491)	2725 (78.1)	766 (21.9)
QRBD (n = 3357)^*^	2524 (75.2)	833 (24.8)
CLIA (n = 3491)	1110 (31.8)	2381 (68.2)

^*^106 samples had equivocal result and 28 samples with result not available/ULOQ/Retest/CV out of range/ < 7.99, thereafter they were excluded

**Table 4:** Diagnostic accuracy of QRBD and CLIA test kit in reference to WANTAI and level of agreement

Test Name		WANTAI			Test accuracy of QRBD (95% CI)	Total agreement	Kappa (SE, 95% CI)
		Positive	Negative	Total			
QRBD	Positive	2482 (98.3)	42 (1.6)	2524 (100.0)	Sensitivity: 94.6% (93.6–95.4%) Specificity: 94.3% (92.3–95.8%)	94.5%	0.84 (SE = 0.018) (95% CI 0.80–0.87)
	Negative	142 (17.1)	691 (82.9)	833 (100.0)			
CLIA	Positive	1098 (98.9)	12 (1.1)	1110 (100.0)	Sensitivity: 40.3% (38.4–42.2%) Specificity: 98.4% (97.3–99.2%)	53.1%	0.22 (SE = 0.011) (95% CI 0.19–0.24)
	Negative	1627 (68.3)	754 (31.7)	2381 (100.0)			

*SE: standard error; CI: Confidence interval

**Table 5:** Diagnostic accuracy of CLIA test kit in reference to QRBD and level of agreement

Test name		QRBD			Test accuracy of CLIA (95% CI)	Total agreement	Kappa (SE, 95% CI)
		Positive	Negative	Total			
CLIA	Positive	1084 (99.2)	8 (0.8)	1092 (100.0)	Sensitivity: 42.9% (41.0–44.9%) Specificity: 99.0% (98.1–99.5%)	56.8%	0.26 (SE = 0.012) (95% CI 0.23 -0.28)
	Negative	1440 (63.6)	824 (36.4)	2264 (100.0)			

^*^SE = standard error, CI = Confidence interval

**Table 6:** Serolo-positivity percentage based on the vaccination status as per the Wantai, QRBD and CLIA test

Test	Vaccinated (n = 371)			Unvaccinated (n = 3117)
	Total	Single dose (n = 284)	Two doses (n = 87)	
Wantai	292 (78.1%)	207 (72.9%)	85 (97.7%)	2431 (77.9%)
QRBD	269 (76.8%) (n = 350)^#^	190 (71.9) (n = 264)^$^	79 (91.6%) (n = 86)^@^	2253 (75.1%) (n = 3001)^θ^
CLIA	151 (40.7%)	102 (35.9%)	48 (55.2%)	958 (30.7%)

^#^Among 371 vaccinated, 9 sample had equivocal result, 12 samples had result not available/ULOQ/Retest/CV out of range/ < 7.99, thereafter they were excluded

^$^Among 284 getting single dose vaccine, 9 sample had equivocal result, 11 samples had result not available/ULOQ/Retest/CV out of range/ < 7.99, thereafter they were excluded

^@^Among 87 getting two doses vaccine, 1 sample had result not available/ULOQ/Retest/CV out of range/< 7.99, thereafter they were excluded

^θ^Among 3117 unvaccinated, 97 sample had equivocal result, 16 sample had result not available/ULOQ/Retest/CV out of range/< 7.99 and 3 participants had unknown history of vaccination, thereafter they were excluded

## Discussion

We assessed the concordance of the three test kits in this population based seroepidemiological study. There was a strong agreement beyond chance between the WANTAI and the QRBD kit (kappa = 0.83) [7]. The total agreement was also high (92.3%) reflecting a high level of concordance.

The diagnostic accuracy of QRBD test in respect to Wantai test was high. Whereas for the CLIA it was low. There was high level of false negative result by CLIA which were positive by Wantai and QRBD. For the CLIA test kit, the total agreement and the kappa value was low in reference to both WANTAI (total agreement = 53.1%, kappa = 0.22) as well as QRBD (total agreement = 56.6%, kappa = 0.26). This can be due to the difference in the target protein of the CLIA and other two tests. The CLIA test captures the antibody against the nucleo-capsid protein (NP) of the virus which is used for the genomic packing while replication [11]. Whereas the other two tests captures the receptor binding domain of spike protein which is used for the binding with the host cell receptor and is a potential target of all the vaccines [12]. Though the WANTAI captures total antibody (IgA + IgG + IgM) and the QRBD captures the IgG only, strong agreement was found. This may be due to the higher proportion of IgG and the longer duration of IgG persists both in natural infection as well as in vaccinated individuals [13, 14]. There was structural and functional difference between the two protein as well as the expression in respect to the antibody production. Therefore the concordance of CLIA test with the other two tests were poor. In a study done by Liu et al. significantly low positive rate was found by tests detecting antibody against nucleocapsid protein [6]. There was inherited difference of the three tests regarding the ability to capture the type of antibody. In spite of this, the evidence of this study helped us to get a further understanding of prevalence obtained through these tests and the degree of agreement. Therefore, the sero-prevalence data always necessitates the understanding of the tests criteria for a clearer picture of the pandemic scenario.

Our study included samples from both urban and rural area. Larger sample size was another strength of this study. The selected age group was also wider having participants from very young to elderly participants.

We did not assess in respect to known serum sample from RTPCR confirmed participants. The urban resettlement area was purposively chosen. Moreover it didn’t represent the whole urban Delhi population.

## Conclusion

The variable level of the diagnostic accuracy and agreement reflects the diagnostic capabilities of different serological assessment. Serological tests are used in clinical and epidemiological context [8, 9]. Therefore, choosing an appropriate test and interpretation of the serology data should be based on such evidences.

## Data Availability

The data and materials is not available publicly. It can be obtained upon reasonable request to the corresponding author.

## Abbreviations

SARS-CoV-2: Severe Acute Respiratory Syndrome Coronavirus-2
WHO: World Health Organization
COVID: Coronavirus Disease
ELISA: Enzyme-linked Immunosorbent Assay
Q-RBD: Quantitative-Receptor Binding Domain
CLIA: Chemiluminescence Assay
AIIMS: All India Institute of Medical Sciences
NCR: Delhi National Capital Region
THSTI: Translational Health Science and Technology Institute
OD: Optical Density
ICMR: Indian Council of Medical Research
